# Implication of human herpesviruses in oncogenesis through immune evasion and supression

**DOI:** 10.1186/1750-9378-9-3

**Published:** 2014-01-20

**Authors:** Kenneth Alibek, Yeldar Baiken, Ainur Kakpenova, Assel Mussabekova, Samal Zhussupbekova, Madina Akan, Bolat Sultankulov

**Affiliations:** 1 Nazarbayev University, 53 Kabanbay Batyr Avenue, Astana 010000, Kazakhstan; 2 National Medical Holding, 2 Syganak Street, Astana 010000, Kazakhstan

**Keywords:** Herpesviruses, Cancer, Immune system, Antigen presentation, Natural killer cells, Cytokine

## Abstract

All human herpesviruses (HHVs) have been implicated in immune system evasion and suppression. Moreover, two HHV family members, i.e. EBV and KSHV, are recognised as oncogenic viruses. Our literature review summarises additional examples of possible oncogenic mechanisms that have been attributed to other HHVs. In general, HHVs affect almost every cancer-implicated branch of the immune system, namely tumour-promoting inflammation, immune evasion, and immunosuppression. Some HHVs accomplish these effects by inhibiting apoptotic pathways and by promoting proliferation. Mechanisms related to immunosupression and low grade chronic inflammation could eventually result in the initiation and progression of cancer. In this article we open a discussion on the members of *Herpesviridae*, their immune evasion and suppression mechanisms, and their possible role in cancer development. We conclude that discerning the mechanisms of interplay between HHV, immune system, and cancer is essential for the development of novel preventative and therapeutic approaches for cancer treatment and prophylaxis.

## Introduction

The human herpesviruses include herpes simplex viruses (HSV-1 and HSV-2), varicella zoster virus (VZV), Epstein-Barr virus (EBV), cytomegalovirus (CMV), human herpesvirus 6 and 7 (HHV-6, HHV-7) and Kaposi’s Sarcoma-associated herpesvirus (KSHV). Herpesviruses have acquired the unique ability to exploit a number of immune evasion strategies. These strategies enable herpesviruses to persist in a latent state within host cells, and then reactivate under certain conditions. The significance of herpesvirus research is enhanced by accumulating data that associate these viruses with certain types of cancer [1]. Some of the members of *Herpesviridae*, such as EBV and KSHV, are officially recognized as carcinogens [1]. The role of CMV is well-established in brain tumours, with evidence pointing to several oncogenic mechanisms [2]. Other members of the Herpesviridae family are associated with a malignant phenotype, but their role in carcinogenesis remains unclear and requires further investigation. There are several direct carcinogenic mechanisms exploited by herpesviruses, including inhibiting apoptosis and tumour suppressor pathways, promoting the oncogenic microenvironment, facilitating cellular migration, metastasis, angiogenesis, and inducing mutagenesis (see Table 1).

**Table 1:** Direct oncogenic mechanisms exploited by human herpesviruses

**Human herpesvirus**	**Associated cancers**	**Direct oncogenic mechanisms**
**Herpes simplex virus 1 and 2 (HSV-1 and HSV-2)**	HSV1 was detected in benign and malignant thyroid tumours [3], associated with prostate cancer, melanoma incidence in both men and women [4], associated with cervical cancer [5]	Induces unscheduled DNA synthesis [6]. In lytically infected cells, both HSV-1 and HSV-2 possess an anti-apoptotic activity, specifically HSV-2 protein ICP10PK, which inhibits apoptosis through activation of Ras/Raf-1/MEK/ERK pathway [7]
**Human herpesvirus 3, or Varicella zoster virus**	Leukaemia, lymphoma, skin cancer, benign and malignant breast tumours [8]	VZV ORF12 activates AP1, a transcription factor that upregulates cellular proliferation [9]. VZV ORF63 inhibits apoptosis by IE63 protein [10]
**Human herpesvirus 4, or Epstein-barr virus (EBV)**	Nasopharyngeal Carcinoma, Burkitt’s Lymphoma and Hodgkin’s lymphoma, to lesser extent	RASSF1A promoter methylation, p16 homozygous deletions andmethylation [11,12]
	HIV-positive CNS lymphomas and hypopharyngeal and laryngeal tumours [13-15], rarely post-transplant smooth muscle tumours [16]	
**Human herpesvirus 5, or human cytomegalovirus**	Glioblastoma (90% association), lymphoma, nasopharyngeal cancer, cervical cancer, Kaposi’s sarcoma (KS), colorectal carcinoma, prostate cancer, skin cancer, astrocytomas [17,18]	Inducing cell cycle progression, activation cell motility and migration, induces VEGF expression, inhibiting DNA damage repair, inducing chromosomal aberrations, inhibiting apoptotic pathways [2]
**Human herpesviruses 6 and 7 (HHV6 and HHV7), or roseoloviruses**	Paediatric lymphoma [19]), acute lymphoblastic leukaemia [20], basal cell carcinoma [21], glioma [22]	Restrains p53 [23], DR7 transform NIH3T3 cells [24], cell cycle arrest at G2/M phase [25], integrate into human chromosome [22]
**Human herpesvirus 8, or Kaposi’s sarcoma associated virus (KSHV)**	Kaposi’s sarcoma, Multicentric castleman disease (MCD), Primary effusion lymphoma (PEL)	vIRF3 and ORF73 (LANA) inhibit p53-mediated cell cycle arrest and apoptosis; K13 (ORF71) inhibits extrinsic death-receptor-mediated apoptosis pathway; ORF16 encodes the viral Bcl-2 protein that inhibits apoptosis and suppresses the cellular autophagy pathway [26]

Another important topic of research is the apparent link between herpesviruses and changes in the immune system. Research has shown that almost every branch of the immune system is affected by herpesvirus proteins, microRNAs, or other products. Clinical evidence shows that herpesviruses lead to immunosuppression and are especially harmful in immunocompromised individuals. There is also evidence linking herpesvirus latency with immunosenescence [27,28]. Considering the importance of immune surveillance in the control of cancer cell growth, one hypothesis is that certain human herpesviruses can lead to cancer initiation and progression by disrupting immune system function and response mechanisms. This review covers the major effects of some human herpesviruses on different branches of the immune system, as well as the mechanisms by which these changes may lead to cancer.

### Overview of cancer immune system evasion

The hypothesis of tumour immunosurveillance was first proposed by Thomas and Burnet in 1957. It stated that the human body employs its natural defences and exhibits immunological resistance to the development of cancer. This immunological response occurs during the early stages of cancer development. The clinically detectable signs of tumour disease can only be seen after the cancerous cells have already evaded the immune response [29]. The following list of observations supports the presence of an immune response against cancerous cells [30,31]:

• Tumour-specific immunity, including cytotoxic T-lymphocytes (CTLs) and antibodies.

• Favourable prognosis in immunocompetent patients.

• Cases of spontaneous regression of certain cancers.

• Strong association between immunodeficiency and cancer.

Furthermore, tumours can escape immunosurveillance by several mechanisms [32]:

• Loss or downregulation of MHC class I molecules.

• Downregulation of tumour antigens.

• Suppressing the immune response by ineffective signals to CTLs.

• Immunosuppressive cytokine release.

The detailed discussion below will show how human herpesviruses have been implicated in cancer initiation and progression using immunosurveillance-based mechanisms.

### Human herpesviruses 1 and 2: the herpes simplex viruses (HSVs)

HSV-1 and HSV-2 have evolved numerous mechanisms to evade host detection and immune responses and usually establish lifelong latency. HSV-1 has been detected in benign and malignant thyroid tumours, while HSV-2 has been found associated with papillary thyroid cancer and the presence of lymph node metastases [3]. Additionally, HSV-2 is associated with prostate cancer, melanoma incidence in both men and women [4], and cervical cancer [5].

HSV-1 and HSV-2 impair the immune system by affecting T-cell receptor signalling. These viruses inhibit the T-cell receptor (TCR)-stimulated formation of a linker required for the activation of a T-cell signalling complex. As a result, TCR-stimulated NF-κB activation and selective TCR-stimulated interleukin-10 synthesis are inhibited, resulting in favoured viral replication and suppressed cellular immunity [33]. HSV has been reported to inhibit the type I interferon response [34], and HSV-1 infected cells can resist T-cell induced apoptosis through expression of the Us5 gene product gJ [35]. The HSV-1 infected cell protein (ICP) 47 blocks the peptide transporter (TAP), preventing access of antigenic peptides into the class I pathway [36]. HSV-1 has also been shown to block CTL-induced Ag-dependent apoptosis [37]. Additionally, infection of human B lymphoblastoid cells (B-LCL) by HSV-1 *in vitro* resulted in decreased ability to induce CD4 + T cell clone proliferation and cytokine secretion through US1 gene expression [38].

### Human herpesvirus 3: varicella zoster virus (VZV)

Herpes zoster is a frequent complication of lymphoreticular malignancy. In immunocompromised patients, varicella may present atypically in the form of disseminated ulcerative or necrotic skin lesions [39]. Also, the duration of replication and virus shedding can be extended, resulting in protracted disease [40]. This complication, like many others, is well-described in advanced stages of leukaemia and lymphoproliferative disorders, particularly after chemotherapy [41].

After initial infection, VZV establishes lifelong latency in the sensory ganglia and reactivates upon immunodeficiency. Some experiments have demonstrated that VZV preferentially infects T cells, particularly CD4 T cells [10,42]. During initial viraemia, infected CD4 T cells cause down regulation of the histocompatibility complex class I expression and allow VZV to evade the immune system [43]. In addition, infected T cells transport VZV via T-cell trafficking from tonsillar tissue to the skin, where VZV replicates and produces cell-free VZV, resulting in highly infectious skin lesions. Uninfected T cells also arrive at the infection sites, become primed with cell-free VZV, and mount a systemic immune response in an effort to control the initial infection [44]. Cellular immunity clearly plays a critical role in controlling VZV reactivation, because the two main factors contributing to zoster incidence are age (most likely reflecting immune senescence) and cellular immune compromise due to disease or iatrogenic cause. Also there is high risk of reactivation of infection in immunocompromised patients, such as those with leukaemia, lymphoma and other malignancies.

Although acyclovir is the standard treatment for herpes virus infections, resistance can develop, causing fatality [45]. VZV may exacerbate the condition of cancer patients and worsen prognosis.

### Human herpesvirus 4 (Epstein-barr virus (EBV))

EBV has continued to attract considerable attention due to its oncogenic properties and its association with a number of human malignancies of both epithelial and lymphoid origin [46,47]. EBV is associated with particular forms of cancers, such as Nasopharyngeal Carcinoma, Burkitt’s lymphoma, and Hodgkin’s lymphoma. To lesser extent, EBV can be found with HIV-positive CNS lymphomas, hypopharyngeal and laryngeal tumours, sinonasal undifferentiated carcinoma, malignant melanoma, and gastric carcinoma [13-15]. Also, the majority of post-transplant smooth muscle tumours are associated with EBV [16].

The initial invasion of EBV to B cells occurs via binding of viral gp350 protein to cellular receptor CD21. Primary infection of naïve B cells in the oropharynx leads to a general infection of the circulating B cells in blood [48]. EBV-infected B cells proliferate in response to the latent viral proteins and RNAs [49]. To enter epithelial cells, viral proteins BMRF-2 and gH/gL interact with cellular transmembrane receptors, which initiates fusion of the viral envelope with the epithelial cell membrane allowing EBV invasion of the epithelial cell [50]. Glycoprotein gp42 acts as a tropism switch and is required for B cell infection, but inhibits epithelial cell infection [51].

Both lytic and latent infections are regulated by the immune response. EBV has evolved different strategies in order to evade immune system and establish latency in memory B cells. For example, some infected B cells deregulate latent gene expression and become long-lived, latently infected memory B cells [27]. Other strategies include disabling immunogenic latent proteins, expressing lytic proteins that interfere with the antigen processing mechanism and the MHC molecule expression in infected cells, and producing viral homologs of human cytokines [52,53].

EBV induces a strong human leukocyte antigen (HLA) class I–restricted, antigen-specific CD8+ CTL response in infected individuals [54]. It is believed that this response plays an important role in regulating the virus during both primary infection and in the long-term carrier state. EBV can impair CD4+ and CD8+ T-cell recognition by a strong, although not complete, HLA I and HLA II down-modulation. This effect is caused by the activity of BNLF2a,4 BILF1 and BGLF5,1,5, which are expressed at different time points during the lytic cycle. Moreover, the virus actively interferes with the effector T-cell action through the viral IL-10 homolog [52].

The transformation of EBV viral particles, such as Epstein-Barr nuclear antigens (EBNAs), latent membrane proteins (LMPs), and Epstein-Barr virus-encoded small RNA (EBERs), from B cells into latently infected lymphoblastoid cell lines ultimately disrupts host genome regulation. The result includes dysregulation of apoptosis, genetic instability, and constant proliferation [11,12]. These effects can be seen in when the virus is in both lytic and latent cycles. In lymphoproliferative disorders expression of only EBNA 1 is revealed in type I latency [55]. Type II latency shows the expression of EBNAs and LMPs [56]. Whereas, in type III latency, all EBNAs and LMPs are expressed mostly associated with EBV-positive post-transplant lymphoproliferative disorders and HIV-associated lymphoproliferative disorders [57].

EBV has been shown to specifically bind to monocytes through a CD21-independent receptor [58]. Also, such interactions resulted in the modulation of cytokine gene expression, e.g., IL-1 and IL-6 production [59], and suppression of the synthesis of TNF-α, a pleiotropic cytokine that induces apoptosis and exhibits anticancer activity.

### Human herpesvirus 5 (human cytomegalovirus (HCMV))

HCMV has been linked to numerous cancer types, including glioblastoma (90% association), lymphoma, nasopharyngeal cancer, cervical cancer, Kaposi’s sarcoma, colorectal carcinoma, prostate cancer, skin cancer, and astrocytomas [18]. HCMV can infect many types of immune cells, including monocytes, macrophages, dendritic cells (DCs), and granulocytes. Neutrophils are the major carrier during an acute infection, while monocytes and macrophages are responsible for dissemination and latent virus reservoirs. Tumour-associated macrophages are infected with HCMV within the glioblastoma [2]. HCMV-infected monocytes fail to differentiate into DCs, thereby inhibiting the antigen presentation process [60].

HCMV affects the immune response in a wide variety of ways. HCMV proteins block antigen presentation and the recognition of CTL and NK cells. A functional IL-10 homolog also inhibits the NK cell response [2]. Viral gene products lead to CTL evasion by interfering with TAP. Expression of antigenic peptides during latency is linked to immune “memory inflation,” leading to immunosuppression and an inefficient response against new antigens [28]. HCMV miRNA (HCMV-miR-UL112-1) helps the host cell to evade NK cell recognition. HCMV also induces B cell-related immune memory inflation via immunodominant epitopes [61]. Additionally, HCMV induces immunosuppressive M2 type macrophage responses. When residing in monocytes, HCMV blocks cytokine-induced macrophage differentiation, leading to impaired phagocytosis [60].

The viral transcript UL111A encodes two transcripts that bear homology to human IL-10 (latent and active), which function to mimic human IL-10. They inhibit pro-inflammatory cytokine production and decrease the expression of MHC I and II, thus exhibiting immunosuppressive properties. In addition, they stimulate proliferation and differentiation of B lymphocytes and monocytes. This phenotype helps the virus to establish primary productive infection and contributes to persistent viral shedding [62]. In addition, HCMV induces IL-6 and TNF-α expression, contributing to oncogenic microenvironment [2].

### Human herpesviruses 6 and 7 (HHV-6 and -7)

Roseoloviruses, HHV6 and HHV7, are linked to many lymphoproliferative cancers as well as other types, including paediatric lymphoma [19], Hodgkin’s lymphoma, non-Hodgkin’s lymphoma, acute leukaemia [63], basal cell carcinoma [21] and glioma [64]. HHV6 is a T-lymphotropic virus. HHV6 is permissive to neural, epithelial and fibroblast cells *in vitro*, but has a particular tropism to immune cells, such as CD4+ T cells, monocytes, macrophages, NK cells, and DCs. HHV6 enters through human CD46 receptor, which is important for T cell response and can induce high proliferation of IL-10 [65]. HHV-6 is then transmitted to CD4+ T cells via monocyte-derived DCs [66]. The virus replicates predominantly in CD4+ T lymphocytes and reduces their proliferation [67]. HHV-6 eventually persists latently in monocytes or macrophage-lineage cells [68]. HHV-7 infects both primary CD4+ T lymphocytes and SupT1 lymphoblastoid T-cell line. This type of infection contributes to cancer development due to an accumulation of cells in gap 2/mitosis (G2/M) phase, polyploidy, and an increased cell size. In HHV7 infected cells due to the cell-cycle regulation, cdc2 is activated, thus preventing cytotoxic T-cells activity and altering immune response [69].

HHV-6 and HHV-7 possess both immunosupressive properties and proinflammatory properties, as indicated by their ability to alter the cytokine expression profile of infected cells. HHV-6A induces IL-18 and IFN*γ* receptors, supresses the apoptotic response-associated cytokines IL-6 and TNF-α, and converts T cells to a Th1 phenotype [64,65]. HHV-6A infection also promotes IL-6, IL-8, and TGF-β production in cultures of astrocytes [64]. Moreover, viral proteins U22, U51 and U83 resemble human chemokines. As an example, U83 can bind to CCR receptors expressed by leukocytes and thus promote leukocyte infiltration [65,68]. Similarly, HHV-7 affects the immunomodulatory cytokines mentioned above. In the supernatants of infected cells, elevated levels of these cytokines were found. On the other hand, the expression of IL-2 was decreased and overall cytokine-induced cellular proliferative responses were down-regulated by this virus [70].

### Human herpesvirus 8 (HHV-8)

The HHV-8 genome contains 86–87 open reading frames (ORFs) or genes, about quarter of which may play a role in modulation of host immune system. Most of these ORFs are predicted to produce proteins that bear homology to host proteins. Thus, HHV-8 has the potential to elaborately evade host immune response [26,71]. HHV-8 is extensively associated with two human malignancies, KS and PEL. Additionally, the virus sequence has been detected in multiple myelomas and angio-immunoblastic lymphomas using PCR [72]. Development of HHV-8-associated cancers is usually linked to immunosuppression [10]. For example, KS occurs about 100 times more frequently in untreated AIDS patients [10,73].

Since HHV-8 is transmitted mainly through saliva, it can infect mucosal-associated macrophages, DCs, lymphocytes and endothelial cells. B cells are the major reservoir for HHV-8 latency, where it resides as an episome and evades host immune responses through several molecular pathways. These molecular pathways can also directly provoke oncogenesis when host immunity is disrupted [10,72,74].

XBP-1s protein, one of the host transcription factors for plasma cell maturation, is also used by HHV-8 for the transcription activation of the lytic switch protein. Thus, HHV-8 appears to have evolved to undergo lytic replication during the differentiation of B cells. Another factor for B cell differentiation is cellular microRNA (miR)-155. HHV-8 encodes its homolog, known as miR-K12-11, which can play role in modulating processes controlled by cellular micro-RNAs [75]. Due to the sequence-specific regulation and non-immunogenic properties of miRNA, arguably that viruses may apply this strategy to improve the cellular environment for viral infection and pathogenesis [76]. The viral interleukin-6 (vIL-6) protein of HHV-8 has mitogenic properties and supports B cell proliferation [77]. It has broader cell specificity than cellular IL-6 because it does not require the gp-80 alpha subunit of the IL-6 receptor in order to bind gp130 and activate IL-6 signal transduction [26,71,78].

HHV-8 inhibits MHC class I antigen presentation through K3 and K5 proteins, which are expressed during lytic cycle and localized at the endoplasmic reticulum. They contribute to increased endocytosis of MHC I molecules, followed by antigen degradation at the proteasome [71]. There is some evidence that HHV-8 escapes the NK cell response by decreasing expression of NKp30, NKp46, and CD161 receptors [73]. The HHV-8 vOX2 protein also alters cellular uptake by inhibiting the phagocytosis of antibody-coated virus particles by neutrophils. vOX2, encoded by K14, mimics cellular CD200 protein, and binds its receptor CD200R with similar affinity [75].

HHV-8 inhibits the complement system through the KSHVcomplement control protein (KCP) encoded in ORF4 [26,79]. KCP is a homolog to RCA proteins that can inactivate C3 convertases, thus preventing complement activation [71]. KCP inactivates C3 convertases by speeding up their decomposition. Two forms of C3 convertase, classical and alternative, contain C4b and C3b components, respectively. KCP can act as a cofactor for proteins that cleave both C4b and C3b. Thus, KCP inhibits complement cascade by two different mechanisms, first by directly contributing to C3 convertase decomposition, and second by serving as a cofactor for cleavage proteins of C4b and C3b [79].

Four of the HHV-8 genes encode for viral interferons (vIRF-1 through 4), which are homologous to cellular interferons. vIRFs repress the immune responses normally mediated by cellular interferons through several mechanisms. First, they prevent transcriptional activation mediated by cellular Ifn. Second, they accelerate degradation of some of the Ifn molecules. Additionally, vIRFs inhibit signalling pathways driven by type I interferons (α and β) and Ifn-λ. Finally, these viral interferons can inhibit transcription and apoptosis induced by p53 and PKR proteins [26,71,72].

In additional to interferon homologs, the HHV-8 genome also contains chemokine homologs. Among the HHV-8 lytic-cycle genes are three chemokines homologs, vCCL-1, vCCL-2 and vCCL-3. They share about 41% amino acid sequence similarity with CC chemokine macrophage inflammatory protein (MIP)-1α [71]. These viral chemokines reduce host antiviral response by acting as Th2-cell receptor agonists [26].

### Overview of immune system-related oncogenic mechanisms exploited by HHVs

Human herpesviruses have developed numerous mechanisms of immune evasion. Some of the effects of HHVs on immune system may have implications in cancer initiation and progression. The information presented above shows that, although each member of *Herpesviridae* family has unique gene products and pathways, there are some similarities in the mechanisms by which they evade the immune system. Table 2 summarises the two major types of tumour-promoting mechanisms: immune suppression and inflammation. It is also clear that both innate and adaptive immune system pathways are impaired by the actions of HHVs. The combination of these mechanisms may potentially initiate or reinforce a cancer phenotype.

**Table 2:** Immune-system related oncogenic mechanisms exploited by HHVs

	**Immune system-related oncogenic mechanism**	**HHV(s) implicated**
**Tumour-promoting immuno-suppression and immune evasion mechanisms**	Inhibition of antigen presentation	HSV-1, HSV-2, VZV, EBV, HCMV, HHV-8
	vIL-10 expression or human IL-10 related immunosuppression	HSV-1, HSV-2, EBV, HCMV, HHV-6, HHV-7, HHV-8
	Inhibition of CTL and NK cell responses	All HHV
	Inhibition of phagocytosis	HCMV, HHV-8
	Inhibition of complement response	KSHV
**Tumour-promoting inflammation**	T and B cell immune memory inflation by the presence of immunodominant epitopes	EBV, HCMV
	IL-6 induction	EBV, HCMV, HHV-6, HHV-7, HHV-8

## Conclusions

Our knowledge of the role that human herpesviruses play in cancer initiation and progression is constantly evolving with continued investigation. Although several direct oncogenic mechanisms have been observed, it is clear that herpesviruses can promote tumour initiation and/or progression using mechanisms related to the immune system function and response. It is important to note that both tumour-promoting inflammation and immunosuppression have been implicated in this process. Based on the findings reviewed in this article, it is clear that nearly all branches of the anti-tumour immune response may be exploited by herpesviruses and their gene products. Further studies are needed to elaborate the role of these mechanisms in carcinogenesis and to apply this knowledge in the development of novel cancer therapies.

## Abbreviations

HSV: Herpes simplex virus; VZV: Varicella zoster virus; EBV: Epstein-barr virus; HCMV: Human cytomegalovirus; KSHV: Kaposi sarcoma-associated herpes virus; CNS: Central nervous system; ORF: Open reading frame; IE: Immediate early; RASSF1A: Ras association (RalGDS/AF-6) domain family member 1; DR7: vIRF, viral interferon regulatory factor; LANA: Latency-associated nuclear antigen; KS: Kaposi sarcoma; MCD: Multiple castleman disease; PEL: Primary effusion lymphoma; CTL: Cytotoxic T cell; MHC: Major histocompatibility complex; TCR: T cell receptor; TAP: Transporter associated with antigen processing; CD: Cluster of differentiation; gp: Glycoprotein; HLA: Human leukocyte antigen; IL: Interleukin; EBNA: Epstein-barr nuclear antigen; LMP: Latent membrane proteins; EBER: Epstein-barr virus-encoded small RNA; TNF: Tumour necrosis factor; UL: Th – T helper, TGF, XBP, vOX2; NK: Natural killer; DC: Dendritic cell; Ifn: Interferon, vCCL, MIP, macrophage inflammatory protein; KCP: KSHV complement control protein.

## Competing interests

The authors declare that they have no competing interests.

## Authors’ contribution*s*

KA, AM, AK, YB, SZ, MA, and BS performed the literature research and wrote the manuscript. All authors read and approved the final manuscript.
